# A Respiratory Sound, Produced by a Tumor of the Chest

**Published:** 1855-03

**Authors:** E. R. Maxson

**Affiliations:** Adams’ Centre, N. Y.


### ART. II.—*A Respiratory Soundro, pduced by a Tumor of the Chest.*

By E. R. Maxson, M. D., Adams' Centre, N. Y.

Mrs. G., about sixty-five years of age, had, at the age of forty-five or fifty, a tumor on the front of the chest, just below the clavicle, to which some application was made, which appeared to arrest its growth; after which she suffered no particular inconvenience from it. She was in the enjoyment of tolerable health for the succeeding twenty years, except frequent attacks of sick headache.

During the summer of 1853, about twenty years after the first appearance of the tumor, her sick headache ceased, and she began to suffer from pain in the right side, extending from the spine along the cervicle and upper dorsal portion forward to the sternum, and extremity of the floating ribs; this last point being the most painful.

This pain, though constant, and increasing, did not prevent her from attending to her household affairs most of the time, till late in the fall, when it began to prevent sleep, and medical aid was sought. Her health had not, however, suffered materially except from the severe pain.

About the first of March following, in consequence of the sickness of her physician, she came under my care for about one month. At first I could but regard the pain as neuralgic, but as there was little or no tenderness of the spine, I could not feel certain as to the cause, unless that portion of the spine, and its nerves, had taken on the irritation that used to develop itself in headache.

Not being able to discover any settled disease, except the pain—and as there was an asthenic condition—I prescribed potassa iodide, grs. v., three times per day as an alterative, and ferri citras, grs. iii., three times per day as a tonic; and directed that she take a nourishing diet. I also applied a narrow blister on that side of the spine, and afterward cups, and anodynes.

Her appetite improved, but the pain continued most of the time.

I soon discovered a slight lateral curvature of the spine, and an apparent fullness of the posterior part of that side of the chest. This, together with the fact that a few particles of blood were raised from the throat or lungs, induced me to make a thorough examination of the chest by auscultation.

From the posterior examination I discovered in the left lung an increased activity in the respiratory sounds, and in the right lung a slow or lengthened inspiration, and two or three distinct interruptions, in each expiration, as though something interrupted, by direct pressure, across the posterior portion of the lung, a little above the middle. Now as I have never heard the sound before, and do not remember to have seen a description of it, and as it was so very distinct, I have named it the Interrupted Expiratory Sound.

This peculiar interrupted expiration suggested to me the possibility of a tumor on the posterior and inner walls of the chest, involving the intercostal nerves, and thus producing the severe and protracted pain in that side of the chest. The fact, too, that the remains of the old tumor under the clavicle had disappeared since the pain commenced, and as the interrupted expiration became more distinct, led me to suggest to her physician, (who had by this time, April 15, recovered and taken charge of his patient,) the possibility of the existence of such a tumor. It was also confirmed by a dull sound, on percussion, of all the lower portion of that side of the chest.

The same treatment was continued, we both thinking that if such was the case nothing better could be done.

As she lived in an adjoining town, I saw nothing more of her till April 26, when I was invited to attend the *post-mortem* examination, she having died rather suddenly, after a slight hæmoptysis, which continued for several hours. I was informed that the action of the heart had been very irregular for several days, and that percussion over the right lung gave a very dull sound, which increased rapidly before death.

On opening the chest the pericardium was found distended with water. The right lung was gorged with blood, or rather hepatized in appearance, especially in its lower portion; and on removing the lung a tumor, of a fibrous character, was found adhering to the pleura, intercostal muscles, and ribs, and reaching from the spine across the posterior cavity of the thorax, and pressing on the lung a little above the middle, had compressed the lung at that point, to about one-half its natural thickness. There was no other morbid appearance except slight adhesion of the pulmonary and costal pleura.

The tumor was at the precise point where I first discovered the peculiar interrupted expiration, which first suggested to me the possibility of its existence. The slight curvature of the spine and prominence of that side cf the chest, indicates the effort which the system made to accommodate the cavity of the thorax to the condition of the suffering lung. The gorged state of the lung was evidently owing to the interruption in the return of the blood past the tumor, in its passage along the pulmonary veins, on its way back to the heart.

The effusion into the pericardium was, doubtless, produced, in part, by the interruption to the free passage of the blood from the heart by the obstruction in the lung. And the severe neuralgic pain along the intercostal nerves, which had been so distressing from the beginning of her illness, was evidently caused by those nerves being involved in the thickening and hardening at the base of the tumor.

My apology for offering the particulars of this case for publication, is the interest I have in bringing this peculiar interrupted expiratory sound before the profession, in hopes that it may serve as an important means of diagnosis in such or similar cases hereafter.

ADAMS' CENTRE, N. Y., Jan. 10, 1855.

